# HLA-DRB1 risk alleles for RA are associated with differential clinical responsiveness to abatacept and adalimumab: data from a head-to-head, randomized, single-blind study in autoantibody-positive early RA

**DOI:** 10.1186/s13075-021-02607-7

**Published:** 2021-09-18

**Authors:** William Rigby, Jane H. Buckner, S. Louis Bridges, Marleen Nys, Sheng Gao, Martin Polinsky, Neelanjana Ray, Vivian Bykerk

**Affiliations:** 1 Dartmouth-Hitchcock Medical Center, 1 Medical Center Dr, Lebanon, NH 03766 USA; 2 Benaroya Research Institute at Virginia Mason, 1201 9th Ave, Seattle, WA 98101 USA; 3 Division of Rheumatology, Hospital for Special Surgery, 535 E 70th St, New York, NY 10021 USA; 4 Bristol Myers Squibb, Avenue de Finlande 4, 1420 Braine-I’Alleud, Belgium; 5 Bristol Myers Squibb, 3401 Princeton Pike, Princeton, NJ 08648 USA

**Keywords:** Abatacept, Adalimumab, Anti-citrullinated protein antibodies, Biological therapy, Arthritis, rheumatoid, Therapeutics

## Abstract

**Background:**

Certain risk alleles associated with autoantibody-positive rheumatoid arthritis (RA) have been linked to poorer prognoses. In patients with autoantibody-positive RA, abatacept shows differential efficacy to tumor necrosis factor inhibitors. Our aim was to investigate the relationship between clinical response to abatacept and to adalimumab and presence of risk alleles encoding human leukocyte antigen *(HLA)-DRB1* shared epitope (SE) in RA.

**Methods:**

In this head-to-head study, biologic-naïve adults with early (≤ 12 months), moderate-to-severe RA and inadequate response to methotrexate (MTX-IR), autoantibody-positive for both anti-cyclic citrullinated peptide 2 and rheumatoid factor, were randomized 1:1 to receive subcutaneous abatacept 125 mg weekly or subcutaneous adalimumab 40 mg every 2 weeks for 24 weeks with stable, weekly oral MTX. An open-label period to 48 weeks followed, during which adalimumab-treated patients were switched to abatacept. Patients were genotyped for *HLA-DRB1* alleles and classified as SE-positive (≥ 1 SE allele) or SE-negative (no SE alleles). Efficacy was assessed at weeks 24 and 48.

**Results:**

Forty patients each received abatacept (9 SE-negative, 30 SE-positive, one unknown) or adalimumab (9 SE-negative, 31 SE-positive). Mean age and disease duration were 46.0 years and 5.5 months, respectively. At week 24, a greater percentage of abatacept patients achieved 50% improvement in ACR criteria (ACR50) compared with adalimumab patients (73% vs 45%, respectively) and estimate of difference (95% confidence interval [CI]), 28 (5, 48). In SE-positive patients, ACR50 estimate of difference (95% CI) was 32 (7, 55). During the open-label period, responses were sustained in the abatacept non-switch group and showed trends toward further improvement in the adalimumab-to-abatacept switch group at week 48, in both the overall and the SE-positive subpopulation. No new safety signals were identified.

**Conclusions:**

In MTX-IR patients with early, autoantibody-positive RA, abatacept resulted in numerically higher efficacy responses versus adalimumab after 24 weeks, with more pronounced treatment differences in SE-positive patients. After 48 weeks, responses were sustained in patients who continued abatacept while those who switched to abatacept showed further clinical improvement, overall, and in SE-positive patients. This supports co-stimulation blockade as an effective treatment strategy for patients with early, autoantibody-positive RA, particularly among SE-positive patients.

**Trial registration:**

NIH US National Library of Medicine, NCT02557100. Registered on September 23, 2015.

**Supplementary Information:**

The online version contains supplementary material available at 10.1186/s13075-021-02607-7.

## Background

In rheumatoid arthritis (RA), autoreactive T cells are activated, mediating the production of pro-inflammatory cytokines and autoantibodies. Downstream inflammatory events, including the recruitment of neutrophils and production of cytokines such as tumor necrosis factor (TNF)-α, result in the development of synovitis and, ultimately, joint destruction [1]. Certain risk alleles are associated with autoantibody-positive (i.e., positive for autoantibodies such as rheumatoid factor [RF] and/or anti-citrullinated peptide antibodies [ACPAs]) RA and may portend a less favorable disease prognosis [2, 3].

Several treatment options are available in RA, including conventional synthetic, advanced biologic (b), and targeted synthetic disease-modifying antirheumatic drugs (DMARDs). The full impact of the different mechanisms of action among biologics is poorly understood, and head-to-head comparisons are lacking. Treatment may alter the immune profiles of patients with RA in a manner dependent on the mechanism of action of the particular biologic.

The human leukocyte antigen (HLA) system (the major histocompatibility complex [MHC] in humans) encodes cell-surface molecules which present antigenic peptides to T cells [4]. Antigen-presenting cells (APCs) express MHC Class II cell-surface receptors [5], such as HLA-DR [4]. The *HLA-DRB1* alleles commonly associated with RA encode amino acid sequences referred to as the shared epitope (SE); these sequences are located at amino acids 70–74 (e.g., QKRAA, QRRAA, RRRAA) in the third hypervariable region of the β-chain (particularly DRB^pos-11^) [2, 6, 7]. The SE is present in up to 80% of anti-cyclic citrullinated peptide 2 (anti-CCP2)-positive Caucasian patients with RA [8–10], in whom it is associated with increased binding of citrullinated peptides [11]. Alleles encoding the SE have lower prevalence among patients with RA of African descent, although they are still enriched in patients with RA compared with healthy individuals [12, 13]. In SE-positive patients, some citrullinated peptides bind to SE-positive HLA-DRB1 with higher affinity than their unmodified forms, leading to T-cell activation and the subsequent generation of antibodies to citrullinated peptides [14]. In patients with joint complaints, *HLA-DRB1* SE alleles strongly associated with anti-CCP-positive RA but not anti-CCP-negative RA [8]. ACPAs, in turn, are associated with a less favorable clinical course and prognosis of RA [15, 16].

The notion that the SE may predict responsiveness to abatacept arose from the AMPLE (*A*batacept versus adali*M*umab com*P*arison in bio*L*ogic-naïv*E* RA patients with background methotrexate [MTX]) study. This study examined the effects of two therapies with differing mechanisms of immune modulation. In this phase IIIb, multinational, prospective, randomized, single-blind study, abatacept, and adalimumab demonstrated similar efficacy and safety profiles after 2 years of treatment in patients with a mean RA duration of < 2 years [17, 18]. AMPLE data showed that high serum levels of anti-CCP2 antibodies were associated with an improved response to abatacept but not to adalimumab [19].

Based on the AMPLE study [19], and given the known association of ACPAs with *HLA-DRB1* SE status [20], in the present study, we hypothesized that the presence of the SE might define a subset of patients with early, autoantibody-positive RA whose immunopathophysiology leads to enhanced clinical responses to treatment with a T-cell co-stimulatory modulator, such as abatacept, versus a TNF inhibitor, such as adalimumab. Abatacept is a selective co-stimulation modulator that blocks the interaction between CD80/CD86 on APCs and CD28 on T cells. Relative to adalimumab, abatacept interferes more directly with T-cell activation and the persistent loss of immune tolerance in SE-positive patients, evidenced by the increased production of ACPAs [20]. Consistent with our hypothesis, in a retrospective Japanese observational study, clinical efficacy of abatacept was significantly higher in SE-positive versus SE-negative patients [21]. Moreover, SE positivity was found to be predictive of response to treatment with abatacept, but not tocilizumab, which inhibits the interleukin-6 pathway [22]. Reports on the association of the SE with response to TNF inhibitors have been mixed. A post hoc genetic analysis of patients with early RA from a randomized controlled study suggested an association between the SE copy number and adalimumab response [23]. To the contrary, another study of several agents (infliximab, etanercept, and adalimumab) in a large UK-wide cohort found no association between treatment response and the SE, although a non-TNF inhibitor control group was not included [24].

To test our hypothesis, this “Early AMPLE” study aimed to prospectively explore, in greater detail, characteristics of the immune profile of patients with early, autoantibody-positive RA, and inadequate response to MTX (MTX-IR) treated with abatacept versus adalimumab, each in combination with MTX. The main differentiating factors between Early AMPLE and the original AMPLE study are the shorter mean disease duration (5.5 months vs 1.7–1.9 years, respectively), higher baseline anti-CCP2 positivity (100% vs 76.4% of patients, respectively), and RF positivity (100% vs 76.5% of patients, respectively). A pre-specified objective of this study was to prospectively investigate the relationship between the clinical efficacy of two different treatments in relation to the presence or absence of the *HLA-DRB1* SE alleles known to be associated with disease severity.

## Methods

### Study design and interventions

**Fig. 1:**
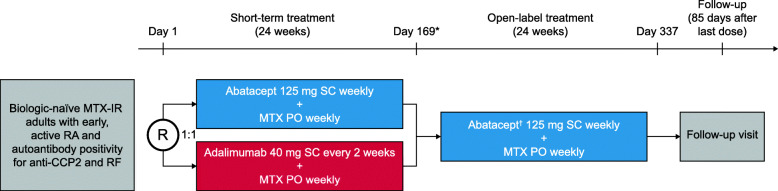
Study design. Asterisk indicates patients could discontinue after the short-term treatment period and continue to follow-up. Dagger indicates patients receiving adalimumab switched to abatacept at week 28, following a 6-week washout period (4-week biologic-free window). CCP2, cyclic citrullinated peptide 2; MTX, methotrexate; MTX-IR, inadequate response to methotrexate; PO, orally; R, randomization; RA, rheumatoid arthritis; RF, rheumatoid factor; SC, subcutaneous

Early AMPLE was a phase IV, multinational, exploratory, randomized, head-to-head, single-blind study (ClinicalTrials.gov, NCT02557100). Between November 2015 and March 2019, patients were enrolled at 23 sites; 19 sites in the USA, three sites in Mexico, and one site in Canada. Patients were randomized 1:1 to receive subcutaneous (SC) abatacept 125 mg weekly or SC adalimumab 40 mg every 2 weeks, each for 24 weeks (single-blind period; Fig. 1); SE status of participants was not known at the time of randomization. At week 28, adalimumab-treated patients were switched to open-label SC abatacept 125 mg weekly following a 6-week washout period (4-week biologic-free window; switch group); abatacept-treated patients continued treatment in an open-label manner (non-switch group) for 24 weeks (open-label period). Both groups received stable, weekly, maximum tolerated doses of oral MTX (15–25 mg) throughout the study.

The random assignment of patients was carried out via a central randomization system. A randomization block (*n* = 4) design was used without stratification. Randomization schedules were generated and kept by the Randomization Group within Drug Supply Management of Bristol Myers Squibb. Double blinding of the study drugs was not feasible due to logistical barriers around re-packaging adalimumab, as well as the expected differences in injection-site reactions. Patients were not blinded with regard to their study drug, but the clinical assessors of joints, disease activity, and judged adverse event (AE) causality were blinded to treatment assignment. The study was conducted in accordance with the ethical principles of the Declaration of Helsinki [25], the International Conference on Harmonization Guidelines for Good Clinical Practice, and local regulations. At each site, an institutional review board or independent ethics committee approved the protocol, consent forms, and any other written information provided to patients or their legal representatives.

### Patients

The study aimed to explore treatment differences between small patient cohorts using specific parameters; therefore, the target population was narrowly defined to minimize inter-patient heterogeneity and ensure that the clinical phenotype under study was representative of SE-positive RA. Eligible patients were male or female, ≥ 18 years of age, and had a diagnosis of early RA, defined as symptoms of RA that started ≤ 12 months prior to screening and satisfied the American College of Rheumatology (ACR)/European League Against Rheumatism (EULAR) 2010 criteria for the classification of RA [26] at some point during that time. Key eligibility criteria included autoantibody positivity for anti-CCP2 (with levels > 3× the upper limit of normal [ULN] of 10 U/mL, i.e., > 30 U/mL) and RF. Moreover, eligible patients had to be treatment naïve with regard to any biologic, targeted, or conventional DMARDs other than MTX. An inadequate response to MTX was required, as characterized by a Disease Activity Score in 28 joints using C-reactive protein (DAS28 [CRP]) ≥ 3.2 at screening and ≥ 3 tender and ≥ 3 swollen joints at screening and at randomization, despite treatment with MTX for ≥ 12 weeks, with a stable dose of oral MTX for ≥ 4 weeks prior to randomization (i.e., MTX-IR). Patients continued receiving a stable MTX regimen throughout the study, with no option to increase the dose. In case of intolerance or adverse reaction, the dose of MTX could be lowered to the minimum of 7.5 mg weekly. Oral corticosteroids were permitted at a stable dose equivalent to ≤ 10 mg prednisone daily for ≥ 4 weeks prior to randomization. An intra-articular injection of corticosteroids (≤ 40 mg methylprednisolone or triamcinolone equivalent) was permitted into one joint or divided into two joints only once during the study period, except within 42 days of the week 24 or week 48 study visit; any joint receiving an intra-articular injection was counted as “active” for the remainder of the study. Inhaled and topical corticosteroid use was permitted. Non-steroidal anti-inflammatory drugs and analgesics, such as paracetamol/acetaminophen and tramadol, were permitted at a stable dose, but not within 12 h before a joint assessment.

### Study endpoints and assessments

This was an exploratory study with only exploratory objectives (pre-specified in the study protocol); as such, no primary or secondary objectives were defined. Clinical efficacy endpoints were pre-specified and assessed at week 24 as the proportion of patients with 20/50/70% improvement in ACR criteria (ACR20/50/70 responders), DAS28 (CRP) remission status (defined as < 2.6), and Simplified Disease Activity Index (SDAI) remission status (defined as ≤ 3.3) in the abatacept and adalimumab treatment groups. Adjusted mean changes from baseline in DAS28 (CRP), SDAI, and Clinical Disease Activity Index (CDAI) scores were assessed in each treatment group. In addition, week 24 data were analyzed by SE status (positive or negative) based on *HLA-DRB1* genotype. Treatment differences between abatacept and adalimumab in SE-positive and SE-negative patients were assessed for ACR20/50/70 responders and DAS28 (CRP), CDAI (remission defined as ≤ 2.8), and SDAI remission achievers at week 24. Exploring the relationship between clinical efficacy and the presence of known risk alleles, including the HLA Class II SE, was one of the pre-specified exploratory objectives. Clinical efficacy was also assessed to week 48. Analyses were performed for weeks 24 (presented previously) [27] and 48; all data presented (week 24 and 48 time points) here are from the final, more comprehensive week 48 database lock.

### Key laboratory assessments

Serum samples were collected for the measurement of RF and anti-CCP2 levels. RF was analyzed using Tina-quant® RF II assay according to the manufacturer’s instructions (Q2 Solutions). The positivity cut-off value was 14 U/mL. Anti-CCP2 level was determined using the EliA™ immunofluorescence assay according to the manufacturer’s instructions. Samples with anti-CCP2 levels above upper limit of detection were further diluted to obtain quantitative values (Q2 Solutions). The validated positive cut-off value for the EliA™ anti-CCP2 test of 10 U/mL was used as the ULN. Therefore, a cut-off value of > 30 U/mL was used as the inclusion criterion of anti-CCP2 > 3× ULN. *HLA-DRB1* genotypes were determined using targeted next-generation sequencing on whole blood samples (LabCorp, Burlington, North Carolina, USA). Patients were considered SE-positive if they had ≥ 1 of the following *HLA-DRB1* alleles [2, 6, 22, 28–30]: *01:01, *01:02, *01:05, *04:01, *04:04, *04:05, *04:08, *04:09, *04:10, *04:13, *04:16; *04:19, *04:21, *04:35, *04:66, *10:01, *14:02, *14:06, *14:09, *14:13, *14:17, *14:19, *14:20, and *14:21.

### Statistical analysis

As this was an exploratory study, no formal sample size or power calculation was performed. Enrollment of approximately 40 randomized patients per treatment group was planned. The as-treated analysis population was employed for all analyses, including those of baseline characteristics and clinical efficacy and safety, and included all patients who had received ≥ 1 dose of study medication.

Baseline demographic and disease characteristics were analyzed descriptively. Nominal *p* values are provided for the comparison of CRP, RF concentration, and the anti-CCP2 status of the SE-negative versus SE-positive group. Estimates of adjusted mean change in efficacy variables were from a repeated measure mixed model that included baseline value, treatment group, time, and time by treatment group interaction, unless noted otherwise. Missing values in the efficacy responder analyses were imputed as non-responders. Safety was analyzed descriptively throughout the study and up to 56 days after the last dose of study drug.

## Results

### Patient disposition and baseline characteristics

Of 117 enrolled (screened) patients, 81 fulfilled the protocol-defined eligibility criteria and were randomized; of these, 80 received at least one dose of study therapy: 40 with abatacept and 40 with adalimumab. In the abatacept treatment group, nine patients were SE-negative, 30 were SE-positive (of whom 23 had one SE allele and seven had two SE alleles), and one had unknown SE status due to loss of their blood sample during transportation. In the adalimumab treatment group, nine patients were SE-negative and 31 were SE-positive (of whom 22 had one SE allele and nine had two SE alleles). No abatacept-treated patients discontinued during the 24-week short-term treatment period of the study; four patients (10%) discontinued in the adalimumab group. Reasons for discontinuation were AEs (*n* = 1), patient request (*n* = 1), death (*n* = 1; sudden death), and loss to follow-up (*n* = 1). During the open-label treatment period up to week 48, three patients (8%) in the non-switch group and one patient (3%) in the switch group discontinued. Reasons for discontinuation were loss to follow-up (*n* = 2) and other (*n* = 1) in the non-switch group, and patient request (*n* = 1) in the switch group.

Baseline characteristics were well balanced across treatment groups (Table 1). Overall, the mean (standard deviation [SD]) age, disease duration, and DAS28 (CRP) were 46.0 (14.4) years, 5.5 (2.6) months, and 5.2 (1.1), respectively. Mean (SD) CRP, anti-CCP2, and RF levels were 13.4 (24.8) mg/L, 1017.4 (1391.5) U/mL, and 132.1 (119.0) U/mL, respectively. This balance, including DAS28 (CRP), was maintained between SE-positive and SE-negative patients (Supplementary Table S1) with the exception that SE-positive patients, compared with SE-negative patients, had significantly higher mean (SD) levels of CRP (16.1 [27.8] vs 4.8 [3.5] mg/L; *p* = 0.0029), anti-CCP2 (1216.6 [1525.1] vs 368.1 [433.6] U/mL; *p* = 0.0002), and RF (148.5 [130.1] vs 78.2 [44.1] U/mL; *p* = 0.0006), respectively. In SE-positive patients, mean (SD) CRP, anti-CCP2, and RF levels were, respectively, 15.1 (19.4) mg/L, 1186.9 (1202.7) U/mL, and 154.8 (139.1) U/mL in those treated with abatacept; and 17.0 (34.4) mg/L, 1245.4 (1803.3) U/mL, and 142.5 (122.7) U/mL in those treated with adalimumab. In SE-negative patients, mean (SD) CRP, anti-CCP2, and RF levels were, respectively, 5.9 (3.5) mg/L, 389.8 (363.9) U/mL, and 92.8 (32.1) U/mL in those treated with abatacept, and 3.7 (3.4) mg/L, 346.3 (515.7) U/mL, and 63.7 (51.1) U/mL in those treated with adalimumab.

**Table 1:** Baseline demographic and disease characteristics in the overall population (as-treated analysis)

Characteristic	Abatacept + MTX (***n*** = 40)	Adalimumab + MTX (***n*** = 40)	Total (***N*** = 80)
**Demographic characteristics**			
Age, years	47.2 (12.2)	44.7 (16.3)	46.0 (14.4)
Weight, kg	75.8 (16.8)	67.9 (16.9)	71.8 (17.2)
Female, *n* (%)	29 (72.5)	31 (77.5)	60 (75.0)
Race, *n* (%)			
White	36 (90.0)	36 (90.0)	72 (90.0)
Black/African American	4 (10.0)	1 (2.5)	5 (6.3)
Asian	0	2 (5.0)	2 (2.5)
Other	0	1 (2.5)	1 (1.3)
Ethnicity, *n* (%)*			
Hispanic/Latino	4 (10.0)	6 (15.0)	10 (12.5)
Non-Hispanic/Latino	16 (40.0)	14 (35.0)	30 (37.5)
**Disease characteristics**			
Disease duration, months	5.4 (2.4)	5.5 (2.9)	5.5 (2.6)
Tender joint count, 68 joints	18.7 (16.1)	19.7 (15.2)	19.2 (15.6)
Swollen joint count, 66 joints	13.6 (12.7)	13.1 (8.9)	13.3 (10.9)
Tender joint count, 28 joints	11.5 (7.9)	12.9 (6.8)	12.2 (7.3)
Swollen joint count, 28 joints	10.0 (6.8)	10.1 (5.6)	10.0 (6.2)
Patient pain assessment, VAS (100 mm)	64.8 (19.0)	64.3 (24.2)	64.5 (21.6)
HAQ-DI	1.3 (0.8)	1.4 (0.8)	1.3 (0.8)
Patient global assessment, VAS (100 mm)	61.3 (20.2)	57.2 (25.3)	59.3 (22.8)
Physician global assessment, VAS (100 mm)	56.9 (20.0)	60.8 (18.2)	58.9 (19.1)
CRP, mg/L	12.7 (17.3)	14.0 (30.7)	13.4 (24.8)
RF-positive, *n* (%)	39 (97.5)	39 (97.5)	78 (97.5)
RF level, U/mL	139.5 (123.8)	124.7 (115.0)	132.1 (119.0)
DAS28 (CRP)	5.2 (1.1)	5.2 (1.2)	5.2 (1.1)
SDAI	34.5 (16.1)	36.1 (15.3)	35.3 (15.6)
CDAI	33.3 (15.2)	34.7 (13.8)	34.0 (14.4)
Anti-CCP2 level, U/mL	991.8 (1104.8)	1043.1 (1643.3)	1017.4 (1391.5)
**Concomitant medication**			
NSAIDs, *n* (%)	27 (67.5)	31 (77.5)	58 (72.5)
DMARDs			
MTX, *n* (%)	40 (100.0)	40 (100.0)	80 (100.0)
MTX dose, mg/week	16.6 (3.4)	17.4 (4.1)	17.0 (3.8)
Biologics, *n* (%)	0	0	0
Corticosteroids			
Oral and/or injectable, *n* (%)	24 (60.0)	22 (55.0)	46 (57.5)
Oral, *n* (%)	24 (60.0)	22 (55.0)	46 (57.5)
Oral dose, mg/day	6.5 (2.9)	6.4 (2.8)	6.4 (2.8)

*CCP2* cyclic citrullinated peptide 2, *CDAI* Clinical Disease Activity Index, *CRP* C-reactive protein, *DAS28* Disease Activity Score in 28 joints, *DMARD* disease-modifying antirheumatic drug, *HAQ-DI* Health Assessment Questionnaire-Disability Index, *MTX* methotrexate, *NSAID* non-steroidal anti-inflammatory drug, *RF* rheumatoid factor, *SD* standard deviation, *SDAI* Simplified Disease Activity Index, *VAS* visual analog scale

Data are mean (SD) unless otherwise indicated. Baseline is day 1 of the study. The mean oral dose of corticosteroids (prednisone or prednisone equivalent) and the mean dose of MTX include only patients who have taken at least one dose of oral corticosteroids or MTX, respectively

*US patients only

### Clinical efficacy

**Fig. 2:**
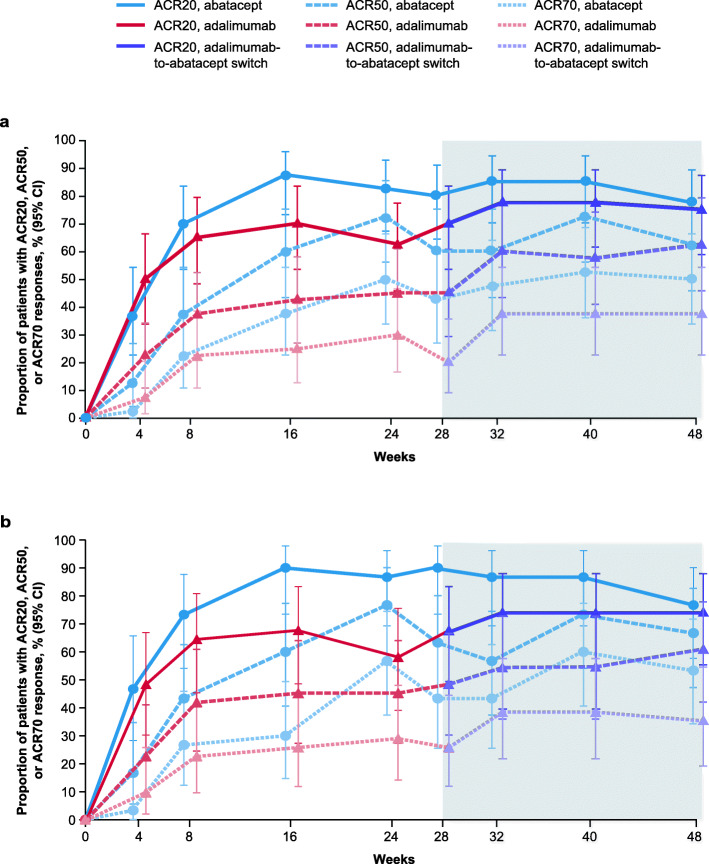
Proportion of patients with ACR responses over time **a** overall and **b** in the SE-positive subpopulation (as-treated analysis). Missing values were imputed as non-responders. Error bars represent 95% CI. ACR, American College of Rheumatology; ACR20/50/70, 20%/50%/70% improvement in ACR criteria; CI, confidence interval; SE, shared epitope. **b** Adapted from Rigby W, et al. EULAR Congress 2020; 4 June 2020; poster THU0160 (with permission of the authors)

**Fig. 3:**
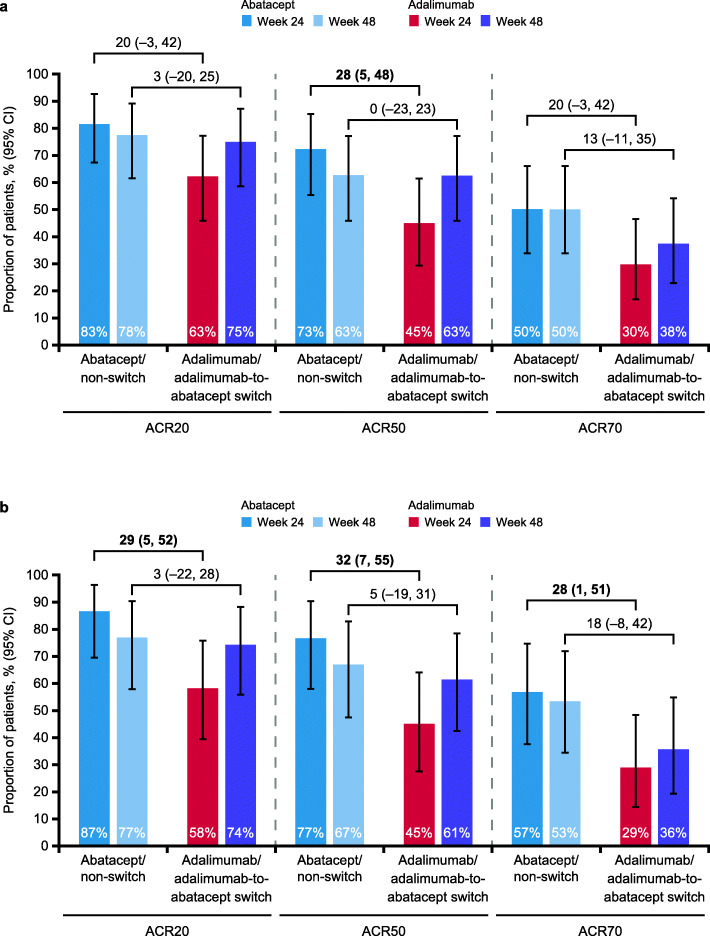
ACR responses at weeks 24 and 48 (as-treated analysis) **a** overall and **b** in the SE-positive subpopulation. Bolded values denote significance. ACR, American College of Rheumatology; ACR20/50/70, 20%/50%/70% improvement in American College of Rheumatology criteria; CI, confidence interval; SE, shared epitope. Figure adapted from Rigby W, et al. EULAR Congress 2020; 4 June 2020; poster THU0160 (with permission of the authors)

Numerically higher responses were seen across all efficacy measures among abatacept- versus adalimumab-treated patients at week 24 (Figs. 2a and 3a; Table 2). Notably, the 95% confidence interval (CI) for estimated treatment differences for ACR50 response did not include zero. Responses were sustained throughout the open-label period to week 48 in the non-switch group across all efficacy measures (Figs. 2a and 3a; Table 2); in the switch group, efficacy responses generally improved over time during the open-label period to week 48. At week 48, ACR20/50 response rates and DAS28 (CRP) remission rates were similar between treatment groups and the ACR70 response rate was still somewhat higher in the non-switch versus the switch group.

**Table 2:** Clinical outcomes at weeks 24 and 48 in the overall population (as-treated analysis)

Clinical outcome	Week 24			Week 48		
	Abatacept + MTX (***n*** = 40)	Adalimumab + MTX (***n*** = 40)	Estimate of difference for abatacept vs adalimumab (95% CI)	Abatacept non-switch (***n*** = 40)	Adalimumab-to-abatacept switch (***n*** = 40)	Estimate of difference for non-switch vs switch (95% CI)
**ACR responses, % (95% CI)**						
ACR20	83 (67, 93)	63 (46, 77)	20 (−3, 42)	78 (62, 89)	75 (59, 87)	3 (−20, 25)
ACR50	73 (56, 85)	45 (29, 62)	**28 (5, 48)**	63 (46, 77)	63 (46, 77)	0 (−23, 23)
ACR70	50 (34, 66)	30 (17, 47)	20 (−3, 42)	50 (34, 66)	38 (23, 54)	13 (−11, 35)
**DAS28 (CRP)**						
Adjusted mean change from baseline (95% CI)	−2.6 (−2.9, −2.3) *m = 39*	−2.4 (−2.7, −2.1) *m = 34*	−0.2 (−0.7, 0.2)	−2.7 (−3.0, −2.5) *m = 36*	−2.9 (−3.2, −2.6) *m = 33*	0.1 (−0.3, 0.6)
Remission, % (95% CI)*	55 (39, 71)	30 (17, 47)	**25 (2, 46)**	48 (32, 64)	50 (34, 66)	−3 (−25, 20)
**SDAI**						
Adjusted mean change from baseline (95% CI)	−28.2 (−30.5, −25.9) *m = 39*	−26.0 (−28.4, −23.6) *m = 34*	−2.2 (−5.5, 1.2)	−28.5 (−30.8, −26.2) *m = 36*	−30.3 (−32.7, −27.9) *m = 33*	1.7 (−1.6, 5.1)
Remission, % (95% CI)^†^	43 (27, 59)	23 (11, 39)	20 (−3, 42)	43 (27, 59)	28 (15, 44)	15 (−8, 37)
**CDAI**						
Adjusted mean change from baseline (95% CI)	−27.3 (−29.6, −25.0) *m = 39*	−25.0 (−27.4, −22.5) *m = 35*	−2.3 (−5.7, 1.0)	−27.8 (−30.1, −25.5) *m = 36*	−29.2 (−31.6, −26.8) *m = 33*	1.4 (−2.0, 4.7)
Remission, % (95% CI)^‡^	43 (27, 59)	23 (11, 39)	20 (−3, 42)	40 (25, 57)	30 (17, 47)	10 (−13, 32)

*ACR* American College of Rheumatology, *ACR20/50/70* 20/50/70% improvement in American College of Rheumatology criteria, *CDAI* Clinical Disease Activity Index, *CI* confidence interval, *CRP* C-reactive protein, *DAS28* Disease Activity Score in 28 joints, *MTX* methotrexate, *SDAI* Simplified Disease Activity Index

Bolded values denote significance. *m* is the number of patients with baseline and post-baseline measurements. Missing values were imputed as non-responders

*Remission = DAS28 (CRP) < 2.6

^†^Remission = SDAI ≤ 3.3

^‡^Remission = CDAI ≤ 2.8

**Fig. 4:**
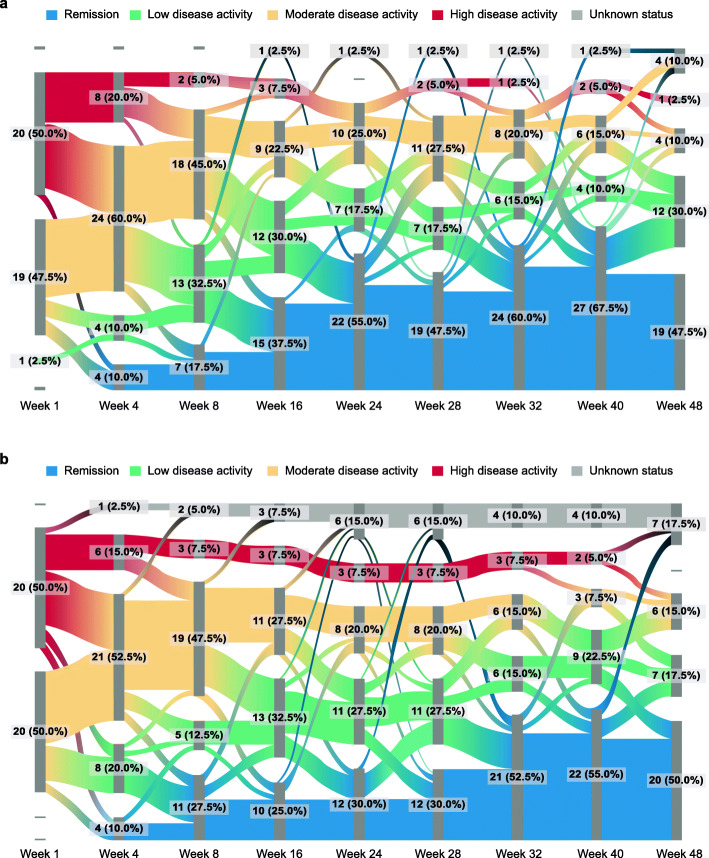
Patient flow per DAS28 (CRP) categories over time (as-treated analysis) **a** abatacept non-switch and **b** adalimumab-to-abatacept switch. *n* = 40 for both groups. CRP, C-reactive protein; DAS28, Disease Activity Score in 28 joints. Figure adapted from Rigby W, et al. EULAR Congress 2020; 4 June 2020; poster THU0160 (with permission of the authors)

The time course of individual patient responses in achieving specific DAS28 (CRP) categories over time to week 48 is depicted using a Sankey flow diagram in Fig. 4. Similar to the main analysis, a numerically higher proportion of patients in the abatacept arm achieved DAS28 (CRP) remission by week 24 versus the adalimumab arm. During the open-label period, further increases in the proportion of patients achieving DAS28 (CRP) remission were seen in the switch group, while these rates remained stably elevated in the non-switch group.

Consistent with observations in the overall population, the SE-positive subpopulation showed numerically greater efficacy responses with abatacept versus adalimumab at week 24 (Figs. 2b and 3b; Supplementary Table S2). Notably, the 95% CI for estimated treatment differences for ACR20/50/70 responses and DAS28 (CRP) remission at week 24 did not include zero (Fig. 3b; Supplementary Table S2). CDAI remission was achieved by 43% and 19% of SE-positive patients treated with abatacept and adalimumab, respectively, at week 24; the estimated treatment difference (95% CI) was 24 (− 2, 46; Supplementary Table S2). SDAI remission was achieved by 43% and 19% of SE-positive patients treated with abatacept and adalimumab, respectively, at week 24; the estimated treatment difference (95% CI) was 24 (− 2, 46; Supplementary Table S2). Due to the small sample size, further stratification of the SE-positive subpopulation, by copies of SE alleles, was not conducted. As observed for the overall population, responses seen in the SE-positive subpopulation in the single-blind period were sustained throughout the open-label period to week 48 in the non-switch group across all efficacy measures (Figs. 2b and 3b; Supplementary Table S2). In the switch group, efficacy responses generally improved during the open-label period to week 48.

Similar to the overall population, analysis of individual patients’ clinical course data showed that many SE-positive patients achieved DAS28 (CRP) remission and low disease activity status to week 48 in both groups (data not shown).

In the overall and SE-positive populations during the single-blind period, little correlation was observed between baseline anti-CCP2 level and adjusted mean changes from baseline in DAS28 (CRP) and SDAI score (data not shown).

No clear treatment difference was observed in SE-negative patients at weeks 24 or 48 (Supplementary Table S2). However, due to the small sample size (*n* = 9 per arm), the findings in this subpopulation should be interpreted cautiously.

### Safety

No new safety signals were identified during the study period (Table 3). During the 24-week single-blind period, in each treatment group, the proportion of patients reported to have experienced one or more related AE (abatacept, 12 [30%]; adalimumab, 11 [28%]) was similar. A related serious AE (SAE) of varicella infection was reported in a single adalimumab-treated patient (3%); this was the only related SAE in the study. During the single-blind treatment period, the AE most frequently reported was upper respiratory tract infection, in three (8%) and 14 (35%) patients in the abatacept and adalimumab groups, respectively.

**Table 3:** Safety summary

	Week 24		Week 48
	Abatacept + MTX (***n*** = 40)	Adalimumab + MTX (***n*** = 40)	Cumulative abatacept population(***n*** = 76)
Deaths	0	1 (2.5)*	0
SAEs	1 (2.5)	2 (5.0)	4 (5.3)
Treatment-related SAEs	0	1 (2.5)^†^	0
Discontinued due to SAEs	0	1 (2.5)	0
AEs	22 (55.0)	28 (70.0)	44 (57.9)
Treatment-related AEs	12 (30.0)	11 (27.5)	17 (22.4)
Discontinued due to AEs	0	1 (2.5)	0

*AE* adverse event, *MTX* methotrexate, *SAE* serious adverse event

Data are *n* (%). Week 24 database lock was used for week 24 data

SAEs include hospitalizations for elective surgical procedures. Treatment-related AE or SAE defined as AE or SAE with related or missing relationship to study medication

*Sudden death

^†^Varicella infection

Among patients who received ≥ 1 dose of abatacept (cumulative abatacept population) including the switch group, AEs were reported in 58% (44/76) of patients; 22% (17/76) of patients had AEs related to study drug (Table 3). SAEs were reported in 5% (4/76) of patients; none were deemed study drug-related. No discontinuations due to AEs were reported.

## Discussion

Data from several earlier randomized clinical trials of abatacept in RA, as well as real-world clinical evidence, suggest that patients with RA who have high titers of ACPA early in their disease course demonstrate greater benefit from abatacept than from treatments with a mechanism of action other than T-cell co-stimulatory blockade [17–19, 31–35]. We conducted this Early AMPLE study to further explore this observation and identify any novel characteristics that further define the features of an RA subpopulation that could potentially derive additional benefit from treatment with abatacept over a TNF inhibitor such as adalimumab. Higher ACR responses were seen with abatacept versus adalimumab after 24 weeks of treatment, supporting prior findings that patients with early autoantibody-positive RA respond better to abatacept [17–19]. During the open-label period, the ACR responses seen during the single-blind period were sustained to week 48 in the abatacept non-switch group; in the adalimumab-to-abatacept switch group, ACR responses generally improved over time to week 48. In addition, analysis of individual patients’ clinical course data showed trends toward further improvement in efficacy response (DAS28 [CRP]) in the adalimumab-to-abatacept switch group at week 48. To further investigate the response to abatacept, we prospectively explored the relationship between the clinical efficacy of abatacept and adalimumab regarding the presence or absence of SE in an MTX-IR, early, autoantibody-positive RA patient population. The vast majority of patients randomized in the Early AMPLE study were SE-positive and showed a numerically higher ACR response to abatacept versus adalimumab compared with the overall population at week 24, indicating that the beneficial effect of abatacept in this population is driven by the SE-positive patient subpopulation. Findings in the SE-positive subpopulation during the open-label period were similar to those from the overall population. Thus, SE status may not only provide a basis for assessing the genetic risk of developing RA and the prognosis regarding expected disease progression but may also influence the choice of treatment in early autoantibody-positive RA. To our knowledge, this is the first study to prospectively show that the presence (or absence) of an HLA epitope associated with the severity and rate of progression of RA may contribute to the differential responsiveness of patients to disease-modifying biologics with differing mechanisms of action.

These results have parallels with findings from the original AMPLE study [17]. The clinical response to abatacept versus adalimumab in this early autoantibody-positive RA population (mean disease duration of 5.5 months) was generally greater than in the original AMPLE study (mean disease duration of 1.7–1.9 years) [17]. At week 24 of AMPLE, 40.3% (95% CI, 34.9, 45.6) of patients treated with abatacept had an ACR50 response [17, 18] compared with 72.5% (95% CI, 56.1, 85.4) seen in this early autoantibody-positive RA population. The clinical response to adalimumab and the safety results were consistent between the two studies and were also consistent with the known profiles of abatacept and adalimumab [17].

Previous studies have shown greater efficacy of abatacept versus other agents in the treatment of ACPA-positive patients with RA [19, 35–37]. In AMPLE, baseline anti-CCP2 positivity was associated with a better response to both abatacept and adalimumab; however, patients with the highest anti-CCP2 antibody concentrations (1060–4894 AU/mL) had better clinical responses to treatment with abatacept compared with adalimumab [19]. Similarly, in the AVERT (*A*ssessing *V*ery *E*arly *R*heumatoid Arthritis *T*reatment; ClinicalTrials.gov: NCT011472726) study, a higher proportion of anti-CCP2-positive versus anti-CCP2-negative patients achieved remission after 1 year of treatment with abatacept with background MTX [36]. In a US-based clinical practice setting, greater efficacy was seen with abatacept, but not TNF-α inhibitors, in anti-CCP2-positive versus anti-CCP2-negative patients with RA [35]. In a meta-analysis of 19 studies, anti-CCP positivity was associated with better EULAR responses in patients with RA receiving abatacept but not in those receiving a TNF inhibitor [37].

The findings of this study are consistent with previous evaluations of the impact of SE status on the clinical efficacy of abatacept. In a retrospective observational study of 72 patients with RA (47 SE-positive and 25 SE-negative), clinical efficacy of abatacept (as per retention rates over 60 months, change in SDAI scores at week 24, and proportion of patients with SDAI remission at week 24) was significantly greater among SE-positive versus SE-negative patients [21]. In an earlier study, SE positivity was found to be associated with response to treatment with abatacept but not to tocilizumab [22]. We postulate that the greater efficacy of abatacept among SE-positive versus SE-negative patients may be related to the mechanism of action of abatacept on T cells. The SE enhances the presentation of citrullinated self-epitopes by HLA, thereby increasing the number of autoreactive T cells [38–40]. This T-cell activation pathway requires a second signal, the co-stimulatory signal, which is blocked by abatacept [41–43]. By blocking co-stimulation, abatacept downregulates the citrullinated epitope-mediated and enhanced T-cell activation that would otherwise occur in SE-positive patients. Therefore, direct modulation of T-cell co-stimulation with abatacept may contribute to the finding of higher efficacy of this drug in SE-positive patients.

This analysis has some important strengths and limitations. The head-to-head study design provides robust and direct comparative data that, if confirmed in a larger study, could assist physicians in making treatment decisions. This link between efficacy outcomes and *HLA-DRB1* risk alleles was tested prospectively, and suggests that the presence of the SE appears to orient the inflammatory process of RA in a direction that is more susceptible to inhibition by abatacept than adalimumab. Notably, this does not correlate with ACPA titers but rather reflects the underlying pathophysiology associated with the SE. The study also provides evidence favoring use of precision medicine in the clinical management of RA; that is, using SE status as a biomarker predictive of greater responsiveness to abatacept early in the course of RA. This prospective analysis of the effect of SE positivity on the efficacy of bDMARDs in the treatment of early autoantibody-positive RA adds to the overall body of evidence available for the two agents studied.

Limitations of this study include its exploratory design and small sample size. Due to the exploratory nature of the analyses, differences in response rates could not strictly be tested for statistical significance. Also, due to the small sample size, the analysis of the SE-positive subpopulation by copies of SE alleles could not be performed. For the same reason (small sample size), the results for the SE-negative patients must be interpreted with caution. It may be of value to perform additional larger studies in SE-negative populations, as well as in less well-defined patient populations such as those with more established RA. In addition, it should be noted that the treatment difference in the overall population was driven by the SE-positive subpopulation. Lastly, since the majority of participants in the Early AMPLE study were of European ancestry, additional studies inclusive of different ethnicities, where the contribution of the SE may vary, are warranted.

## Conclusion

Consistent with previous studies, numerically higher efficacy responses were seen with abatacept versus adalimumab after 24 weeks of treatment in this MTX-IR, early, autoantibody-positive RA population; the responses were more pronounced among SE-positive abatacept-treated patients. Due to small sample sizes in this exploratory study, the observed differences between the two therapies were not statistically significant (based on overlapping 95% CIs). This was true in particular for the more stringent efficacy measures, and should be confirmed in a larger clinical trial. During the open-label treatment period, efficacy responses were sustained in the abatacept non-switch group and showed trends toward further improvement in the adalimumab-to-abatacept switch group at week 48. Similar trends occurred in the overall and SE-positive subpopulations. These data provide further evidence that patients who are SE-positive and have early, autoantibody-positive RA may benefit to a greater extent from therapies focused on T-cell co-stimulatory blockade of the generation of ACPAs and upregulation of adaptive immunity. Results need to be confirmed in larger clinical trials capable of establishing the extent to which *HLA-DRB1* genotyping is sufficiently sensitive and specific. If confirmed, these findings would support the use of *HLA-DRB1* genotyping, along with CCP2 concentration monitoring, as a precision medicine tool to guide treatment of RA via T-cell co-stimulatory blockade, and as a predictor of abatacept efficacy in patients with early autoantibody-positive RA.

## Supplementary Information

**Additional file 1: Supplementary appendix.** Includes **Supplementary Table S1.** Baseline demographic and disease characteristics by SE genotype (as-treated analysis population) and **Supplementary Table S2.** Clinical outcomes at Weeks 24 and 48 by SE genotype (as-treated analysis).

## Data Availability

Bristol Myers Squibb policy on data sharing may be found at https://www.bms.com/researchers-and-partners/independent-research/data-sharing-request-process.html.

## Abbreviations

ACPA: Anti-citrullinated peptide antibody
ACR: American College of Rheumatology
ACR20/50/70: 20%/50%/70% improvement in American College of Rheumatology criteria
AE: Adverse event
AMPLE: *A*batacept versus adali*M*umab com*P*arison in bio*L*ogic-naïv*E* RA
APC: Antigen-presenting cell
CCP: Cyclic citrullinated peptide
CDAI: Clinical Disease Activity Index
CI: Confidence interval
CRP: C-reactive protein
DAS28: Disease Activity Score in 28 joints
DMARD: Disease-modifying antirheumatic drug
EULAR: European League Against Rheumatism
HAQ-DI: Health Assessment Questionnaire-Disability Index
HLA: Human leukocyte antigen
MHC: Major histocompatibility complex
MTX: Methotrexate
MTX-IR: Inadequate response to methotrexate
PO: Orally
R: Randomization
RA: Rheumatoid arthritis
RF: Rheumatoid factor
SAE: Serious adverse event
SC: Subcutaneous
SD: Standard deviation
SDAI: Simplified Disease Activity Index
SE: Shared epitope
TNF: Tumor necrosis factor
ULN: Upper limit of normal
VAS: Visual analog scale
