# Supplementary material for: HLA-DRB1 risk alleles for RA are associated with differential clinical responsiveness to abatacept and adalimumab: data from a head-to-head, randomized, single-blind study in autoantibody-positive early RA
Source: Arthritis Res Ther. 2021 Sep 18;23:245. doi: 10.1186/s13075-021-02607-7 (PMC8449494; doi:10.1186/s13075-021-02607-7)
Supplement: Supplementary file 1 — Additional file 1: Supplementary appendix. Includes Supplementary Table S1. Baseline demographic and disease characteristics by SE genotype (as-treated analysis population) and Supplementary Table S2. Clinical outcomes at Weeks 24 and 48 by SE genotype (as-treated analysis). [file 13075_2021_2607_MOESM1_ESM.pdf]

Supplementary appendix

Supplementary Table S1 Baseline demographic and disease characteristics by SE genotype (as-treated analysis population)

**Characteristic**	**SE− population (*n* = 18)**	**SE+ population (*n* = 61)**
Age, years	46.2 (13.9)	46.1 (14.6)
Weight, kg	70.6 (18.7)	71.7 (16.6)
Female, *n* (%)	14 (77.8)	46 (75.4)
White, *n* (%)	15 (83.3)	56 (91.8)
Disease duration, months	5.8 (3.2)	5.4 (2.5)
Tender joint count, 28 joints	12.8 (8.3)	12.1 (7.1)
Swollen joint count, 28 joints	9.7 (5.7)	10.2 (6.4)
HAQ-DI	1.2 (0.7)	1.4 (0.8)
Patient global assessment of disease activity, VAS (100 mm)	52.7 (22.6)	61.4 (22.8)
Physician global assessment of disease activity, VAS (100 mm)	57.4 (20.5)	59.4 (18.9)
DAS28 (CRP)	5.0 (1.0)	5.3 (1.2)
SDAI	34.0 (13.8)	35.9 (16.2)
CDAI	33.5 (13.6)	34.3 (14.8)
CRP, mg/L	4.8 (3.5)	16.1 (27.8)
Anti-CCP2, U/mL	368.1 (433.6)	1216.6 (1525.1)
RF, U/mL	78.2 (44.1)	148.5 (130.1)

Data are mean (SD) unless stated otherwise. Baseline is Day 1 of the study. Patients with missing data for SE alleles were excluded from the analysis.

CCP2 cyclic citrullinated peptide 2, *CDAI* Clinical Disease Activity Index, *CRP* C-reactive protein, *DAS28* Disease Activity Score in 28 joints, *HAQ-DI* Health Assessment Questionnaire-

Disability Index, *RF* rheumatoid factor, *SD* standard deviation, *SDAI* Simplified Disease Activity Index, *SE* shared epitope, *VAS* visual analog scale.

Supplementary Table S2 Clinical outcomes at Weeks 24 and 48 by SE genotype (as-treated analysis)

Week 24						
	SE− population (*n* = 18)			SE+ population (*n* = 61)		
**Clinical outcome**	**Abatacept + MTX (*n* = 9)**	**Adalimumab + MTX (*n* = 9)**	**Estimate of difference for abatacept vs. adalimumab (95% CI)**	**Abatacept + MTX (*n* = 30)**	**Adalimumab + MTX (*n* = 31)**	**Estimate of difference for abatacept vs. adalimumab (95% CI)**
**ACR responses, % (95% CI)**						
ACR20	67 (30, 93)	78 (40, 97)	–11 (−57, 39)	87 (69, 96)	58 (39, 76)	**29 (5, 52)**
ACR50	56 (21, 86)	44 (14, 79)	11 (−39, 57)	77 (58, 90)	45 (27, 64)	**32 (7, 55)**
ACR70	22 (3, 60)	33 (8, 70)	−11 (−57, 39)	57 (37, 75)	29 (14, 48)	**28 (1, 51)**
**DAS28 (CRP)**						
Adjusted mean change from baseline (95% CI)	−2.3 (−3.2, −1.4) *m = 8*	−2.5 (−3.4, −1.6) *m = 8*	0.2 (−1.2, 1.5)	–2.8 (–3.1, –2.4) *m = 30*	–2.4 (–2.8, –2.0) *m = 26*	–0.4 (−0.9, 0.2)
Remission, % (95% CI)*	44 (14, 79)	56 (21, 86)	–11 (–57, 39)	57 (37, 75)	23 (10, 41)	**34 (8, 56)**
**SDAI**						
Adjusted mean change from baseline (95% CI)	−24.4 (−31.4, −17.4) *m = 8*	−27.5 (−34.5, −20.5)	3.1 (−6.9, 13.2)	–30.0 (–32.6, –27.4)	–26.4 (–29.2, –23.5)	–3.6 (−7.5, 0.3)

	33 (8, 70)	*m* = 8		*m* = 30	*m* = 26	
Remission, % (95% CI)†		33 (8, 70)	0 (−48, 48)	43 (26, 63)	19 (8, 38)	24 (−2, 46)
**CDAI**						
Adjusted mean change from baseline (95% CI)	−23.9 (−30.5, −17.2)<br>*m* = 8	−26.7 (−32.9, −20.4)<br>*m* = 9	2.8 (−6.4, 12.0)	–28.9 (–31.6, –26.2)<br>*m* = 30	–25.1 (–27.9, –22.2)<br>*m* = 26	–3.9 (−7.8, 0.1)
Remission, % (95% CI)‡	33 (8, 70)	33 (8, 70)	0 (–48, 48)	43 (26, 63)	19 (8, 38)	24 (−2, 46)

Week 48

	**SE− population (*n* = 18)**			**SE+ population (*n* = 61)**		
**Clinical outcome**	**Abatacept non-switch (*n* = 9)**	**Adalimumab-to-abatacept switch (*n* = 9)**	**Estimated treatment difference for non-switch vs. switch (95% CI)**	**Abatacept non-switch (*n* = 30)**	**Adalimumab-to-abatacept switch (*n* = 31)**	**Estimated treatment difference for non-switch vs. switch (95% CI)**
**ACR responses, % (95% CI)**						
ACR20	89 (52, 100)	78 (40, 97)	11 (−39, 57)	77 (58, 90)	74 (55, 88)	2.5 (–22, 28)
ACR50	56 (21, 86)	67 (30, 93)	–11 (−57, 39)	67 (47, 83)	61 (42, 78)	5 (–19, 31)
ACR70	44 (14, 79)	44 (14, 79)	0 (−48, 48)	53 (34, 72)	36 (19, 55)	18 (–8, 42)

**DAS28 (CRP)**						
Adjusted mean change from baseline (95% CI)	−2.8 (−3.2, −2.3) *m = 8*	−3.1 (−3.6, −2.6) *m = 8*	0.3 (−0.4, 1.0)	–2.8 (–3.2, –2.5) *m = 28*	–2.9 (–3.3, –2.6) *m = 25*	0.1 (−0.4, 0.6)
Remission, % (95% CI)*	56 (21, 86)	78 (40, 97)	–22.2 (–65.4, 28.5)	47 (28, 66)	42 (25, 61)	5 (–21, 30)
**SDAI**						
Adjusted mean change from baseline (95% CI)	−30.0 (−31.8, −28.3) *m = 8*	−31.3 (−33.1, −29.6) *m = 8*	1.3 (−1.3, 3.9)	–29.4 (–32.3, –26.5) *m = 28*	–30.8 (–33.9, –27.8) *m = 25*	1.4 (−2.8, 5.7)
Remission, % (95% CI)†	44 (14, 79)	67; (30, 93)	–22 (−65, 29)	43 (26, 63)	16 (6, 34)	**27 (1, 49)**
**CDAI**						
Adjusted mean change from baseline (95% CI)	−30.0 (−31.8, −28.3) *m = 8*	−31.2 (−32.9, −29.4) *m = 8*	1.1 (−1.4, 3.7)	–28.5 (–31.3, –25.6) *m = 28*	–29.6 (–32.6, –26.5) *m = 25*	1.1 (−3.1, 5.3)
Remission, % (95% CI)‡	44 (14, 79)	67 (30, 93)	–22 (−65, 29)	40 (23, 59)	19 (8, 38)	21 (−5, 43)

Bolded values denote significance. Estimates of adjusted mean change and treatment difference are from an ANCOVA model with treatment group and baseline efficacy value as covariates. Missing values were imputed as non-responders.

*Remission = DAS28 (CRP) < 2.6.

†Remission = SDAI ≤ 3.3.

‡Remission = CDAI ≤ 2.8.

ACR American College of Rheumatology, *ACR20/50/70* 20%/50%/70% improvement in American College of Rheumatology criteria, *ANCOVA* analysis of covariance, *CDAI* Clinical Disease Activity Index, *CI* confidence interval, *CRP* C-reactive protein, *DAS28* Disease Activity Score in 28 joints, *MTX* methotrexate, *SDAI* Simplified Disease Activity Index, *SE* shared epitope.
